# Prognostic Impact of the *MDM2^SNP309^* Allele in Leukemia and Lymphoma

**DOI:** 10.18632/oncotarget.123

**Published:** 2010-07-27

**Authors:** Sean M. Post, Vinod Pant, Hussein Abbas, Alfonso Quintás-Cardama

**Affiliations:** ^1^Department of Genetics, the University of Texas, M.D. Anderson Cancer Center, Houston, TX, USA; ^2^Department of Leukemia, the University of Texas, M.D. Anderson Cancer Center, Houston, TX, USA

**Keywords:** p53, MDM2, SNP309, non-Hodgkin's lymphoma, acute myeloid leukemia, chronic lymphocytic leukemia

## Abstract

A T-to-G germline single nucleotide polymorphism in the promoter region of MDM2 (SNP309) has been reported to markedly accelerate tumor formation in humans suggesting that it may represent a powerful cancer predisposing allele. Since its first description in 2004, a large number of retrospective analyses involving a wide variety of human malignancies have been reported, showing conflicting results regarding the impact of *Mdm2^SNP309^* status on cancer risk and response to cancer therapy. Here, we appraise the available information on the effect of *Mdm2^SNP309^* in lymphoma and leukemia and discuss the factors that likely account for the conflicting results observed in the studies reported to date.

## INTRODUCTION

Alterations in numerous tumor suppressor pathways involving oncogene overexpression and loss of tumor suppressors have been demonstrated to be crucial for tumorigenesis. Inactivation of the tumor suppressor p53 or components of the p53 pathway is common during tumor development. *TP53* encodes a transcription factor that activates numerous genes that halt tumorigenesis [1]. Mutations in the *TP53* gene occur in over 50% of human cancers [2]. Additionally, some tumors have an ablated p53 pathway yet lack *TP53* mutations, suggesting alterations to other components in the p53 pathway occur during tumorigenesis [2]. One key component of the p53 pathway is *Mdm2*. This gene is amplified or overexpressed in a large number of human cancers that retain wild type *TP53* [3, 4]. *Mdm2* encodes an E3 ubiquitin ligase that negatively regulates p53 protein stability and transcriptional activity [5]. These data demonstrate that decreased p53 activity, resulting from mutations in the *TP53* gene or alterations in *Mdm2* significantly impact tumor development. Data derived from mouse studies have shown that a fine tuned regulation of *Mdm2* levels is necessary to maintain proper p53 homeostasis and therefore p53-mediated tumor suppression, which suggests that modest changes in *Mdm2* levels may have an important impact on tumor development.

Recently, a functional T to G single nucleotide polymorphism (SNP) in the human *Mdm2* gene (^*SNP309G*^) has been identified [6]. This SNP enhances the binding of the transcription factor Sp1 to the *Mdm2* promoter which in turn results in increased *Mdm2* mRNA levels and thereby lower p53 levels. Of importance to cancer development, the *Mdm2^SNP309G^* allele has been associated with an increased cancer risk in some human tumors that express wild type p53 [7-9]. However, some reports have failed to show a correlation between the *Mdm2^SNP309G^* G allele and cancer risk [10, 11]. The impact that *Mdm2^SNP309G^* has on cancer risk is supported by the findings that patients diagnosed with Li-Fraumeni syndrome (LFS), a syndrome resulting from inherited germline mutations in p53, that are also homozygous for the *Mdm2^SNP309G^* allele develop tumors significantly earlier than patients with LFS lacking this polymorphism [6, 12]. These data suggest that increased *Mdm2* levels resulting from the presence of the *Mdm2^SNP309G^* allele may further down modulate an already deficient p53 pathway.

Relatively little is known regarding the impact that subtle genetic modifiers have on tumorigenesis. Examination of large cohorts of patients carrying the *Mdm2^SNP309^* allele suggests that subtle changes to the p53 pathway may have a pronounced impact on tumorigenesis. Several caveats hamper a proper interpretation of such clinical studies, including the fact that they are retrospective in nature, with all the biases associated with such type of analyses, and the fact that they involve patients from different ethnic backgrounds, therefore not accounting for the potential impact that other gene modifiers (e.g. other SNPs) may have on the p53 pathway. Certainly an unbiased prospective analysis of patients with cancer is warranted to definitely delineate the impact of the *Mdm2^SNP309^* allele on cancer risk and response to therapy. The development of mouse models mimicking the human *Mdm2^SNP309^* allele may advance significantly our understanding of the impact that subtle genetic differences may have in the regulation of the p53 pathway. Alterations in the p53 pathway have been reported in an important fraction of patients with leukemia or lymphoma. In this paper we appraise the available information on the impact of the *Mdm2^SNP309^* allele in lymphomagenesis and leukemogenesis.

## IMPACT OF THE *MDM2^SNP309^* ALLELE ON LEUKEMOGENESIS

The tumor suppressor *TP53* is mutated in more than 50% of all human solid tumors and in approximately 30% of patients with leukemia. Notably, p53 has been shown to play a critical role in hematopoiesis. Fluctuations in p53 levels and activity result in drastic consequences to the hematopoietic compartment, as demonstrated in mouse models with haploinsufficiency of its negative regulators Mdm2 and Mdm4 [13, 14]. The *Mdm2^SNP309^* allele has been associated with attenuation of p53 activity and early onset of human cancers [6]. Extrapolation of these results to examine the impact of the *Mdm2^SNP309^* allele on leukemogenesis has rendered multiple studies reporting conflicting results. Three major types of leukemia, chronic lymphocytic leukemia (CLL), childhood acute lymphoblastic leukemia (ALL) and acute myeloid leukemia (AML) have been primarily investigated with regards to *Mdm2^SNP309^* status and outcome.

CLL is the most frequent leukemia in the western hemisphere with an incidence rate of 4/100000. The incidence rates in men are nearly twice as high as in women. CLL is characterized by the accumulation of mature CD5^+^, CD19^+^ B-lymphocytes in the hematopoietic system [15, 16]. Several prognostic factors have been linked to a poor prognosis in patients with CLL. Among them, the presence of unmutated immunoglobulin *(Ig)-V_H_* gene and the expression of CD38 and Zap70 are the ones that have shown more robust results as risk factors in clinical trials [15]. p53 plays a critical role in regulating tumor growth and p53 status has been shown to be critical for survival of patients with CLL. p53 inactivation has been documented in patients with CLL primarily due to 17p13 deletion and point mutations. Patients with CLL exhibit a deletion of 17p13 in approximately 5–7% in early stage disease and in 25–40% in advanced and/or refractory disease [17]. Patients with the 17p13 chromosomal abnormality exhibit very aggressive disease, poor response to chemotherapy, and a very poor prognosis [18]. In addition, *TP53* mutations can be detected in up to 20-30% of patients with CLL [17], which implies that, in the remaining cases, possibly other alterations in the p53 pathway may be involved in CLL. Interestingly, overexpression of *Mdm2*, a major p53 regulator has been shown in 28% of patients with B-CLL. The well documented functional association of *Mdm2^SNP309^* in human tumors, and the important prognostic role of p53 in CLL, provides the rationale for studying the prognostic and predictive implications of *Mdm2^SNP309^*in patients with CLL.

The very first study investigating the impact of *Mdm2^SNP309^* on leukemogenesis did not find any difference in the mean age at diagnosis between the different *Mdm2^SNP309^* genotype subsets [19]. In addition, the authors did not find any significant association between the *Mdm2^SNP309^* and the p53 codon 72 arginine-to-proline (p53^R72P^) polymorphisms or other prognostic markers such as the mutational status of the *V_H_* gene, CD38 expression and Zap70 expression. However, the limited cohort size in this study (n=83) precluded a robust statistical analysis. In contrast, another study reported that carrying the *Mdm2^SNP309G^* allele was associated with a decreased risk of leukemia in patients of Chinese origin [20]. However, once again a small cohort size and clubbing together different kinds of leukemia patients made the results difficult to interpret.

In the first major systematic study, Gryschenko *et al*. analyzed two different cohorts of patients (n=140 and n=111, respectively) and showed that occurrence of homozygous (G/G) *Mdm2^SNP309^* did not predispose to CLL [21]. However, patients that were either heterozygous (T/G) or homozygous (G/G) at the *Mdm2^SNP309^* loci had significantly shortened treatment free survival (TFS) and overall survival (OS) in comparison to patients with the (T/T) genotype. Multivariate analysis also identified *Mdm2^SNP309^* as an independent prognostic factor for TFS and OS. In contrast, an unbiased analysis of a large cohort of patients with CLL (n=418) did not detect any association of *Mdm2^SNP309^* and time to first treatment and overall survival [22]. Likewise, no apparent correlation between the different *Mdm2^SNP309^* genotypes and Binet stage or *IgVH* mutational status was identified. Another study involving an even larger cohort of patients (n=617) also confirmed these results and could not detect any correlation between *Mdm2^SNP309^* and the other established prognostic markers in CLL [23]. Carrying the *Mdm2^SNP309^* allele did not impact OS.

A recent study from Sweden involving 210 patients with CLL followed for over 19 years reported that the OS of patients with at least one *Mdm2^SNP309G^* allele was significantly shorter than that of patients with two *Mdm2^SNP309^*T alleles [24]. However, the age of onset of B-CLL was similar between the two genotypes. The presence of an *Mdm2^SNP309G^* allele in combination with *TP53* mutations or unmutated *IgV_H_* gene status resulted in an additional risk. This study supported the claim by Gryschenko *et al*. of *Mdm2^SNP309^* as a prognostic marker in concert with *TP53* mutations and unmutated *IgV_H_* status. However, these data are in conflict with Kaderi *et al* and Zenz *et al*, which could be explained by the difference in patient cohort composition in these studies. Differences in the number of patients with unmutated *IgV_H_* gene and a higher proportion of advanced Binet stage patients may have accounted for such discrepant results. Additionally, unlike Gryschenko *et al*., the authors did not observe any reduction in TFS in patients carrying two *Mdm2^SNP309T^* versus two *Mdm2^SNP309G^* alleles, suggesting that *Mdm2^SNP309^* mainly influences the outcome of therapy. This contention is intriguing particularly in light of data reported by Saddler *et al*. showing that treatment with nutlin-3a, a drug that disrupts the interaction between *Mdm2* and p53, efficiently induces apoptosis of B-CLL cells *in vitro* [25]. Similar results were also reported by Seyfried *et al* [26]. Moreover, a recent report analyzing a small cohort of patients with CLL (n=75) in conjunction with studies involving the *TCL1* mouse model, an established mouse model of B-CLL, showed that miR34a, a p53 downstream pro-apoptotic target gene expression correlates with *Mdm2^SNP309^* status [27]. *Mdm2^SNP309G^*/^*G*^ or *Mdm2^SNP309T/G^* patients with wild type p53 have a lower expression of miR34a and lower TFS. In aggregate, these data suggest that *Mdm2^SNP309T/G^* or *Mdm2^SNP309G^*/^*G*^ genotypes possibly alleviate the sensitivity to chemotherapy regimens by suppressing the p53 pro-apoptotic activity.

In addition to CLL, studies have also focused on ALL, a leading cause of childhood cancer. ALL accounts for 25-30% of all diagnoses in infants below 1 year of age. p53 mutations are rare in childhood ALL. However, components of the p53 pathway are frequently found mutated in ALL [28]. Indeed, deletion or transcriptional silencing of *p14^ARF^* is frequent in ALL, while *Mdm2* overexpression or silencing of the p53 transcriptional target *p21^CIP1^* has been reported in approximately 50% of patients with ALL and linked with worse prognosis [28]. Analyses of 284 samples obtained from children with ALL revealed that the presence of the *Mdm2^SNP309G^* allele significantly decreased the age of onset for ALL in Caucasian and African-American patients but not Hispanic patients [29]. These findings suggest an ethnic specific effect of *Mdm2^SNP309^* in ALL, which has also been reported in patients with solid tumors. A separate study of 114 children with ALL however could not confirm any statistical correlation between *Mdm2^SNP309^* and age of onset of ALL [30]. Nonetheless, a sex specific effect of the polymorphism on earlier disease onset was identified. Females that are homozygous carriers of the *Mdm2^SNP309G^* allele had a significantly earlier disease onset compared to those carrying two *Mdm2^SNP309T^* alleles. While estrogen has earlier been shown to affect *Mdm2^SNP309^* activity, this observation is quite interesting given the fact that children with ALL have negligible estrogen activity. While it could be hypothesized that fetal estrogen exposure may impact postnatal Mdm2 activity, a more detailed investigation of a larger cohort of patients is required to confirm this hypothesis.

Several studies have addressed the possibility of an association between *Mdm2^SNP309^* and the development of therapy related AML. Therapy related AML arises as a consequence of prior cytotoxic therapy and as many as 10% of patients treated for a first cancer develop this potentially fatal secondary malignancy. Examination of two cohorts of patients (n=80 and n=91) who developed treatment related AML following chemo/radiotherapy regimens for another tumor type did not reveal any significant association between *Mdm2^SNP309^* and the risk of treatment related AML [31]. However, an interactive effect between *Mdm2^SNP309^* and the p53^R72P^ SNP towards increased risk of AML was observed in patients previously treated with chemotherapy. Nevertheless, analysis of data sets from a large group of patients with AML (n=404 and 816) revealed a modest increase in risk of *de novo* AML in patients with an *Mdm2^SNP309G^* background. In agreement with the latter, a study by Xiong *et al*., reported a 3.52-fold increase in AML risk but no association of *Mdm2^SNP309^* with age of onset of AML in a cohort of 231 patients with AML [32]. A recent study reported similar results on 575 children with AML treated on three Children's Oncology Group protocols (CCG 2941/2961/AAML 03P1). This study revealed that patients carrying two copies of the *Mdm2^SNP309G^* allele had an increased susceptibility to AML [33]. However, unlike CLL [21], *Mdm2^SNP309^* status had no apparent effect on treatment outcome in AML.

In conclusion, although these studies present conflicting results, they appear to agree in that the presence of an *Mdm2^SNP309G^* allele correlates with earlier onset of childhood ALL in an ethnicity and sex specific manner but may not correlate with incidence or onset of B-CLL. Additionally, the *Mdm2^SNP309G/G^* genotype increases the risk of *de novo* AML. The presence of the *Mdm2^SNP309G^* allele either in the heterozygous or the homozygous state also significantly impacts the overall survival of patients with CLL or AML. There are several aspects that preclude a clear interpretation of results from these studies. *Mdm2^SNP309^* is a weak modifier of p53 activity and its effect is influenced by sex, age, gender, ethnicity and other environmental factors [8]. Furthermore, CLL, ALL and AML are genetically heterogeneous diseases with varied outcomes in different individuals depending on factors such as age at diagnosis, performance status, and the presence of specific chromosomal abnormalities. Furthermore, there are multiple SNPs in the p53 pathway that could alter p53 function. In fact, the polymorphic p53R72P protein may likewise influence the effect of the *Mdm2^SNP309^* allele. Some of the above studies have taken into consideration the potential interplay between these two SNPs. However, a comprehensive study validating such interplay is lacking. Additionally, mutations or defects in p53 or its modulators such as ATM, Mdm4, or p14^ARF^ may likewise affect p53 function. A multivariate, detailed analysis (metaanalysis) of a larger cohort of a more biologically homogenous population will be more informative in this regard.

Given these complexities, the examination of *Mdm2^SNP309^* status in isolation as a prognostic marker for CLL, ALL or AML and for the intent of designing individualized treatment regimens may not be of great clinical impact. However, a synergistic role of the *Mdm2^SNP309^* allele with other prognostic markers is likely and it may predict for response in patients receiving p53-directed therapy (e.g. nutlin-3a).

## IMPACT OF THE *MDM2^SNP309^* ALLELE ON LYMPHOMAGENESIS

A series of small scale retrospective studies have also been undertaken to address the impact of *Mdm2^SNP309G^* on lymphomagenesis [8, 34-36]. The initial description of the role that *Mdm2^SNP309G^* has on diffuse large B-cell lymphomagenesis (DLBCL) was reported by Bond and colleagues [8]. In this study, the authors demonstrated that women (under the average age of 51, and therefore considered to be premenopausal), who self-identified themselves as being of Ashkenazi Jewish decent, carrying the *Mdm2^SNP309G^* allele were more likely to develop DLBCL than young women harboring two *Mdm2^SNP309T^* alleles. Specifically, women harboring two *Mdm2^SNP309G^* alleles were diagnosed with DLCBL 13-years earlier than women who carried two *Mdm2^SNP309T^* alleles, with an average age of 55 years (ranging from 21 to 87 years) and 68 years (ranging from 55 to 78 years), respectively. In fact, 49% of women carrying *Mdm2^SNP309G^* were diagnosed with DLBCL before the age of 51. This is in direct contrast to premenopausal women carrying *Mdm2^SNP309T/T^*, as no individual younger than 51 years of age presented with DLBCL in this study. It is important to note, that the significant impact of *Mdm2^SNP309G^* on DLBCL development in women of Ashkenazi Jewish descent was lost when patients were diagnosed after the age of 51 (considered menopausal). The fact that young premenopausal women harboring the G nucleotide were more likely to develop DLBCL led the authors to speculate that hormone specific factors might influence this early tumor phenotype. This notion is supported by several observations. First, women exposed to exogenous estrogen have an altered risk in DLBCL development [37]. Secondly, *Mdm2* levels are regulated by estrogen signaling as the *Mdm2* promoter possesses an estrogen response element (ERE) [38]. Interestingly, this ERE lies in a region just upstream of the *Mdm2^SNP309^* polymorphism. Third, the *Mdm2^SNP309G^* allele results in an increased affinity for the transcription factor Sp1 [6]. Several studies have demonstrated that Sp1 activity is further stimulated upon its interaction with hormone receptors such as the estrogen receptor [39]. Taken together, these findings suggest that *Mdm2^SNP309G^* may regulate DLBCL development in a gender-specific mode.

A second report on woman of European Caucasian descent with DLBCL, suggested the *Mdm2^SNP309^* polymorphism has no impact on age of tumor onset, even in premenopausal women (under the age 51) [36]. These results are at variance with those reported by Bond *et al*, as Bittenbring and colleagues were unable to detect a significant impact on DLBCL formation in young women carrying two *Mdm2^SNP309G^* alleles. These differing results highlight the complexity of determining a functional outcome of an individual SNP on a specific tumor type, especially a SNP in a regulatory element such as *Mdm2^SNP309^*. In this report, Bittenbring and colleagues suggest a potential cause for these discordant observations may be due to ethnicity differences between the two studies. This is a potentially plausible explanation as Ashkenazi Jews are more genetically isolated as compared to individuals from Europe as a whole. Indeed, while approximately 25% of Ashkenazi Jews harbor two *Mdm2^SNP309G^* alleles only 14% of central European women carried this allele. This increased prevalence in the *Mdm2^SNP309G^* in the Ashkenazi Jewish community may afford an invaluable opportunity to identify individuals carrying this SNP, and therefore, to ascertain the impact of such genetic variant in lymphomagenesis. Additionally, the differing outcome may be a consequence of a low number of *Mdm2^SNP309G/G^* premenopausal samples used by Bittenbring *et al* (20 premenopausal female patient samples). This fact would warrant that a more exhaustive study be performed. Alternatively and of much more interest, these different results may suggest that other currently undescribed genetic modifiers (especially in the p53 pathway) may also influence the functionality of the *Mdm2^SNP309G^* allele; such as the recently identified SNPs in Mdm4, HAUSP, and/or p53 [40]. This notion is quite plausible as it has been noted that Ashkenazi Jews who are isolated genetically, may harbor additional low penetrant modifiers that could potentially either detract or augment the function of *Mdm2^SNP309G^*. The effect of such compounding polymorphisms on the functionality of *Mdm2^SNP309G^* is unknown and may be different in genetically heterogeneous individuals as compared to more homogenous populations.

## FUTURE DIRECTIONS

To definitely ascertain the impact of the *Mdm2^SNP309^* allele on leukemogenesis and lymphomagenesis, prospective analyses of large cohorts of patients are warranted. Given the inherent biases of all the retrospective studies above-described, which undoubtedly account for the conflicting results, the development of animal models carrying the *Mdm2^SNP309^* allele could certainly be of great interest. Our laboratory has generated two complementary humanized *Mdm2^SNP309^* mouse models (*Mdm2^SNP309G^* and *Mdm2^SNP309T^*) in order to examine the direct impact of this SNP on tumor development [41]. Using these mice we have shown that harboring two *Mdm2^SNP309G^* alleles increases *Mdm2* levels and subsequently decreases p53 as compared to *Mdm2^SNP309T/T^* mice. Furthermore, *Mdm2^SNP309G/G^* mice have an attenuated p53 response following treatment with ionizing radiation. More importantly, *Mdm2^SNP309G/G^* mice (either carrying a p53 germline mutation or not) rapidly develop tumors resulting in shorter survival as compared to *Mdm2^SNP309T/T^* mice. Crossing these mice with the previously reported *TCL1* transgenic mouse [42], an established B-CLL mouse model, or the *BCL6* transgenic mouse, an established model that recapitulated human DLBCL, may provide suitable model systems to investigate the deregulation of the p53 pathway induced by the *Mdm2^SNP309^* allele and its impact on leukemogenesis and lymphomagenesis while prospective studies in patients with these hematological malignancies are conducted.
